# Supplementary material for: Mutation Analysis of Thin Basement Membrane Nephropathy
Source: Genes (Basel). 2022 Oct 2;13(10):1779. doi: 10.3390/genes13101779 (PMC9602179; doi:10.3390/genes13101779)
Supplement: Supplementary file 1 [file genes-13-01779-s001.zip › Tables S1-S4.pdf]

Table S1. *COL4A3* primers.

Oligo name	Sequence	Product (bp)
COL4A3_ex1_FW	GACCGAGCCCTACAAAACC	358
COL4A3_ex1_RV	CTCAGCCTCGCCACTAGC	
COL4A3_ex2_FW	CTGCCGGTTGGGATTTATT	214
COL4A3_ex2_RV	CTGCGGATCTTTCTGCTACC	
COL4A3_ex3_FW	CATGCAAAGAGTCACCATGAA	327
COL4A3_ex3_RV	AGTTCACCATGCACCTAGCA	
COL4A3_ex4-5_FW	TGGGTTTGATGTATGGGTTTC	849
COL4A3_ex4-5_RV	GCATCAGAACACATTTTCTTGC	
COL4A3_ex6-7_FW	CATCTTTTCCCTTGGGTTCA	969
COL4A3_ex6-7_RV	GGCTACCCCAGACTTCCCTA	
COL4A3_ex8-9_FW	ATAGCAGAGAGGGCAGAGCA	1114
COL4A3_ex8-9_RV	GAGAGGCGAAAAGAGAGAGAGA	
COL4A3_ex10-11_FW	AATGGACATTGTTATTAAGTGAGAAG	424
COL4A3_ex10-11_RV	CCTGCTAGGGATCCTTGTGA	
COL4A3_ex12-13_FW	TTGGAACTTTGATGGGTTTAGA	531
COL4A3_ex12-13_RV	CTGGGCTGAGCTGGACTTAG	
COL4A3_ex14-15_FW	GAGGATTTGTCCCACTGTTGA	772
COL4A3_ex14-15_RV	AGTTTGAGTTGGGCATCTTG	
COL4A3_ex16-17_FW	GATTTCCGTATTTGTAAAGTTAGCC	546
COL4A3_ex16-17_RV	TGAAGTCTGTTATTGTAATCCCTTG	
COL4A3_ex18_FW	TTTAATTTGGTTTGTACATGACATC	298
COL4A3_ex18_RV	TGGTTTGACAACAAGGTAAATGTT	
COL4A3_ex19_FW	GGGCCAAAATGAAGAAACTG	276
COL4A3_ex19_RV	TCCTCACCCATGTCAATTCC	
COL4A3_ex20_FW	TTTGTCTAGGTGAGAGTAGGAAAAA	303
COL4A3_ex20_RV	GCCTGGAAACACTTCTAGATCA	
COL4A3_ex21_FW	CCTCTCCATTGTGCAATTTTT	375
COL4A3_ex21_RV	TGATGGCTAAGCTGTGAGGA	
COL4A3_ex22-23_FW	GGCTTCCAATACAAAGATGAGA	885
COL4A3_ex22-23_RV	AAACCACGTTGTGACATCCA	
COL4A3_ex24-25_FW	TGTCTACTTAGAGTTGGCGTTCA	1188
COL4A3_ex24-25_RV	GGCCAACCATTTTTGGAA	
COL4A3_ex26_FW	TTGGAGGATGATTAACCTGTTG	367
COL4A3_ex26_RV	TTGACATATTCTATTCGTTAGAAGCAA	
COL4A3_ex27-28_FW	TCGTATTTTCCGCTATCGTC	1413
COL4A3_ex27-28_RV	GCCTGGCCAGAAAACATT	
COL4A3_ex29-31_FW	TGGTTGAGAGATAAGAGAGTTACTGC	1399
COL4A3_ex29-31_RV	GGACTTGTGGCTGGACAGTT	
COL4A3_ex32_FW	CACTGCAATCCAGCCTAGAA	402
COL4A3_ex32_RV	AAAAGCATAGAGTAAAAAGCGTATG	
COL4A3_ex33-34_FW	TGGAATATTTTGCTTTTCTCACTC	753
COL4A3_ex33-34_RV	AAACACTGGCCCTCACTGTC	
COL4A3_ex35-36_FW	TCCCACGTAGCTGGGATTAC	1111
COL4A3_ex35-36_RV	CCAAGTGGAAAACCAAAACA	
COL4A3_ex37_FW	TTGGGAGAAAGCTTATGTAAAACTG	328

COL4A3_ex37_RV	CAATTTTCTCTGCCCACACC	
COL4A3_ex38_FW	TGCTGAATTCTTACCACATATCC	310
COL4A3_ex38_RV	TGAATAAAAGCAACTGCAGCAT	
COL4A3_ex39-41_FW	CAGACCGTTTCAGTCACTGTTG	1016
COL4A3_ex39-41_RV	ATTGGAACATCTTTAATTCACTAGG	
COL4A3_ex42-43_FW	TCAAGAACTCTAACCCAAGCA	1322
COL4A3_ex42-43_RV	AACATCATGAGAATGGACTAATACAG	
COL4A3_ex44-45_FW	AAAAACTGCTGTGAATTGAGTGA	1018
COL4A3_ex44-45_RV	GCCTCACGGATGTGTTACCT	
COL4A3_ex46-47_FW	CGTGAGGCCATCATCTTCTT	1227
COL4A3_ex46-47_RV	TGGTCTGATCTGAACAACATAAA	
COL4A3_ex48_FW	AAAAACGAGTTTAAGATTTTTGTGTA	390
COL4A3_ex48_RV	AAAATGTATCAGGAGACACTCAAGG	
COL4A3_ex49-50_FW	GCTTCTCTCTAGTAACGATGCTG	609
COL4A3_ex49-50_RV	ATGTTGCACACACGGACAG	
COL4A3_ex51-52_FW	AATTTGAACCCCAATGGACA	1301
COL4A3_ex51-52_RV	GCTTTGTTTTGTGGGGAAAC	

Table S2. *COL4A4* primers.

Oligo name	Sequence	Product (bp)
COL4A4_ex2_FW	TTTGACCCAGAACACAGAACC	277
COL4A4_ex2_RV	TGGAATGATTTGGCTTTTTG	
COL4A4_ex3_FW	GGCTAGTAGGAGCAGCCTCA	262
COL4A4_ex3_RV	CCCAGAGGGTGATTTCTTTG	
COL4A4_ex4_FW	TCATGGAGTCAAAGGTAAACCA	274
COL4A4_ex4_RV	TTTGCCAAGAATCTGGGATTA	
COL4A4_ex5_FW	CCATCTCCAGCCATTTGTTT	403
COL4A4_ex5_RV	AGATCCCACCACTGCATTCT	
COL4A4_ex6_FW	TTGGTTCTTAAATGTTGGCTTTT	500
COL4A4_ex6_RV	TGTGGGGGATTGAAAGTAACA	
COL4A4_ex7_FW	GAATCACAATTGCCATAATTTGAG	324
COL4A4_ex7_RV	ATGCTCCAGGCACACTTGTA	
COL4A4_ex8_FW	GGGTGATAATAATATGGAAGAGGA	336
COL4A4_ex8_RV	TGGTGTATTCAGGGACATTTTG	
COL4A4_ex9_FW	TCCTCAATTTTAGTCAACACCAA	288
COL4A4_ex9_RV	ACTAGGTGAGCCGCACAAAT	
COL4A4_ex10-12_FW	GGGATCACACGCAACTCTTT	905
COL4A4_ex10-12_RV	CAGCCATAAAATTGGGCAGT	
COL4A4_ex13-15_FW	TGACACATAGGATTGGAAGCA	1460
COL4A4_ex13-15_RV	CACTTTTGAGCTTGTGGGACT	
COL4A4_ex16-17_FW	AAATGATGCACTGAGCTGGTT	574
COL4A4_ex16-17_RV	TCTTGAATGATTCCTGGCAATA	
COL4A4_ex18-19_FW	GCACATGATCGTTCTAATACTGG	1194
COL4A4_ex18-19_RV	CACAATCCTAAGGTGATTCCAA	
COL4A4_ex20_FW	ACCTCCAGCTCCGTCTCTTT	421
COL4A4_ex20_RV	CAACCAACTTAGCTCATGAAACA	
COL4A4_ex21-22_FW	AAAGAATGGGGTTGGGAAAG	312
COL4A4_ex21_RV2	CAAATCTGAATGAAATGCCACA	
COL4A4_ex22_FW2	CAATTGTCTGCAAGAAGTGCAT	420
COL4A4_ex21-22_RV	TGCGAGTCAATTAAACCTCTTTC	
COL4A4_ex23_FW	GGAGGGAAGAAGAGAGAGATTCA	264
COL4A4_ex23_RV	TGTATAGAATTGATCTATGGTCACAGG	
COL4A4_ex24_FW	CCAACCCAGAATCAAGGTCA	304
COL4A4_ex24_RV	TGAATTTCAGCTTGGCAAAT	
COL4A4_ex25_FW	TGGACTTTTGCGTGTGTTTC	406
COL4A4_ex25_RV	TGTACACTACTCGGGTGACAGG	
COL4A4_ex26_FW	TTGAGACAGACTCACATTGCAC	266
COL4A4_ex26_RV	TCCATGGTCCCTCAAAACTT	
COL4A4_ex27-28_FW	CGGGATAGGCAACATCCTTA	1108
COL4A4_ex27-28_RV	TGAGGGAAAACACTTGGGTAA	
COL4A4_ex29_FW	GGATGAAAATGTTCACCTCCA	357
COL4A4_ex29_RV	TTCTGGCAGCATCCATAAAA	
COL4A4_ex30_FW	GCTTTACTGTTGTTTAACGCATTG	400
COL4A4_ex30_RV	AGGGAAGGACAAAGCCAGAC	
COL4A4_ex31_FW2	GGGTGGATCACTTGAGGCTA	741
COL4A4_ex31_RV2	CCTTTCCCAAAAGGTGAGAA	

COL4A4_ex32_FW	AAGTGGGAATTTGCGATTTG	336
COL4A4_ex32_RV	GAGCAAAACTCCATCTCAAAAA	
COL4A4_ex33-34_FW	GAATCCTCAATACCTTTGGCTTT	1325
COL4A4_ex33-34_RV	CCTTGGGTGTTGCAGTTTCT	
COL4A4_ex35_FW	CCATAAACGTTACTGTCACCTG	266
COL4A4_ex35_RV	TGCACAAAGGGGTAAGAAAA	
COL4A4_ex36-37_FW	AACGTTATTTTCCTTTCCAATAGA	1303
COL4A4_ex36-37_RV	TGGCACCTACGGAAAAGAGT	
COL4A4_ex38_FW	GACGCTCAGAGCTGTTTGCT	306
COL4A4_ex38_RV	CAGTTTTGCCTCCAAATCTCA	
COL4A4_ex39-40_FW	TGGGAAGTGGTTCCTTTTCA	522
COL4A4_ex39-40_RV	GGGCTGCTTCAGTGCTTCTA	
COL4A4_ex41_FW	CCTTGCTTGCAAGTCATTGAA	379
COL4A4_ex41_RV	TGGAAGGTAGTCACTTTGTTTGC	
COL4A4_ex42_FW	GCTATGCCCTGCATTTTGTT	336
COL4A4_ex42_RV	AAAGGGCTTAAATAATAAGAATGTGC	
COL4A4_ex43_FW	AAATCTCTTTCCACCCTGTGAA	202
COL4A4_ex43_RV	ATCCTTACAGCACCCCATCT	
COL4A4_ex44_FW	GACAAATTTCTGCCTTCATGG	445
COL4A4_ex44_RV	TGCAAAGGGAAAGACATGC	
COL4A4_ex45_FW	GCATTCAGTCAAAGTGGGAAG	333
COL4A4_ex45_RV	TCCAGTTTGGAAAGCCACTT	
COL4A4_ex46_FW	AGTGCCAGAACAGAGGTGCT	366
COL4A4_ex46_RV	AAGGTTAGTGATCATTTAAGGTGCT	
COL4A4_ex47-48_FW	GGCCTCCAGATTTCAGAACA	1188
COL4A4_ex47-48_RV	CCAAAATGAGCACCATGACA	

Table S3. Evaluation of the pathogenicity of the 10 variants identified by Sanger sequencing.

Gene	*COL4A3*	*COL4A4*	*COL4A3*	*COL4A3*	*COL4A4*	*COL4A4*	*COL4A4*	*COL4A3*	*COL4A4*	*COL4A4*
	c.469G>C	c.2510G>C	c.697G>A	c.1229G>A	c.827G>C	c.2573G>A	c.71+1G>A	c.2969_2980del	c.1733G>T	c.904delG
Amino acid change	p.Gly157Arg	p.Gly837Ala	p.Gly233Arg	p.Gly410Glu	p.Gly276Ala	p.Gly858Glu	p.?	p.Ala990_Pro993del	p.Gly578Val	p.Gly302ValfsTer23
Allele Frequency	0.00001604	0.000008189	0	0.00000401	0.00001607	0	0	0	0	0
ACMG classification overall	Uncertain significance	Likely Pathogenic	Likely Pathogenic	Uncertain significance	Uncertain significance	Likely Pathogenic	Pathogenic	Likely Pathogenic	Likely Pathogenic	Pathogenic
**Evidence of Pathogenicity**										
Very Strong - PVS1							Yes			Yes
Strong - PS1										
Strong - PS2										
Strong - PS3										
Strong - PS4										
Moderate - PM1										
Moderate - PM2			Yes			Yes	Yes	Yes	Yes	Yes
Moderate - PM3										
Moderate - PM4								Yes		
Moderate - PM5										
Moderate - PM6	Yes	Yes		Yes	Yes	Yes	Yes	Yes		Yes
Supporting - PP1		Yes							Yes	
Supporting - PP2	Yes	Yes	Yes	Yes	Yes	Yes			Yes	
Supporting - PP3	Yes	Yes	Yes	Yes	Yes	Yes			Yes	
Supporting - PP4	Yes	Yes	Yes	Yes	Yes	Yes	Yes	Yes	Yes	Yes
Supporting - PP5		Yes	Yes				Yes		Yes	

**Key For ACMG categories**	
Very Strong - PVS1	Null variant (nonsense, frameshift, canonical ±1 or 2 splice sites, initiation codon, single or multiexon deletion) in a gene where LOF is a known mechanism of disease
Strong - PS1	Same amino acid change as a previously established pathogenic variant regardless of nucleotide change
Strong - PS2	De novo (both maternity and paternity confirmed) in a patient with the disease and no family history
Strong - PS3	Well-established in vitro or in vivo functional studies supportive of a damaging effect on the gene or gene product
Strong - PS4	The prevalence of the variant in affected individuals is significantly increased compared with the prevalence in controls
Moderate - PM1	Located in a mutational hot spot and/or critical and well-established functional domain (e.g., active site of an enzyme) without benign variation
Moderate - PM2	Absent from controls (or at extremely low frequency if recessive) (Table 6) in Exome Sequencing Project, 1000 Genomes Project, or Exome Aggregation Consortium
Moderate - PM3	For recessive disorders, detected in trans with a pathogenic variant
Moderate - PM4	Protein length changes as a result of in-frame deletions/insertions in a nonrepeat region or stop-loss variants
Moderate - PM5	Novel missense change at an amino acid residue where a different missense change determined to be pathogenic has been seen before
Moderate - PM6	Assumed de novo, but without confirmation of paternity and maternity
Supporting - PP1	Cosegregation with disease in multiple affected family members in a gene definitively known to cause the disease
Supporting - PP2	Missense variant in a gene that has a low rate of benign missense variation and in which missense variants are a common mechanism of disease
Supporting - PP3	Multiple lines of computational evidence support a deleterious effect on the gene or gene product (conservation, evolutionary, splicing impact, etc.)
Supporting - PP4	Patient's phenotype or family history is highly specific for a disease with a single genetic etiology
Supporting - PP5	Reputable source recently reports variant as pathogenic, but the evidence is not available to the laboratory to perform an independent evaluation

Table S4. Evaluation of the pathogenicity of the 3 variants identified by exome sequencing.

Gene	*KANK1*	*NPHS1*	*NUP205*
	c.2896+2T>G	c.2869G>C	c.4799T>A
Amino acid change	p.?	p.Val957Leu	p.Phe1600Tyr
Allele Frequency	0	0.003	0.002
ACMG_classification_overall	Pathogenic	Uncertain significance	Uncertain significance
**Evidence of Pathogenicity**			
Very Strong - PVS1	Yes		
Strong - PS1			
Strong - PS2			
Strong - PS3			
Strong - PS4			
Moderate - PM1			
Moderate - PM2	Yes		
Moderate - PM3			
Moderate - PM4			
Moderate - PM5			
Moderate - PM6	Yes	Yes	Yes
Supporting - PP1			
Supporting - PP2		Yes	Yes
Supporting - PP3		Yes	
Supporting - PP4			
Supporting - PP5			
